# “Obviously, Nothing’s Gonna Happen in Five Minutes”: How Adolescents and Young Adults Infrastructure Resources to Learn Type 1 Diabetes Management

**DOI:** 10.1145/3613904.3642612

**Published:** 2024-05-11

**Authors:** Tian Xu, Emily Jost, Laurel H. Messer, Paul F. Cook, Gregory P Forlenza, Sriram Sankaranarayanan, Casey Fiesler, Stephen Voida

**Affiliations:** Information Science, University of Colorado Boulder, Boulder, Colorado, USA; University of Colorado Anschutz, Medical Campus, Aurora, Colorado, USA; University of Colorado Anschutz, Medical Campus, Aurora, Colorado, USA; University of Colorado Anschutz, Medical Campus, Aurora, Colorado, USA; University of Colorado Anschutz, Medical Campus, Aurora, Colorado, USA; Computer Science, University of Colorado Boulder, Boulder, Colorado, USA; Information Science, University of Colorado Boulder, Boulder, Colorado, USA; Information Science, University of Colorado Boulder, Boulder, Colorado, USA

**Keywords:** diabetes management, hybrid-closed loop systems, context-aware health technology, infrastructuring health practice

## Abstract

Learning personalized self-management routines is pivotal for people with type 1 diabetes (T1D), particularly early in diagnosis. Context-aware technologies, such as hybrid closed-loop (HCL) insulin pumps, are important tools for diabetes self-management. However, clinicians have observed that practices using these technologies involve significant individual differences. We conducted interviews with 20 adolescents and young adults who use HCL insulin pump systems for managing T1D, and we found that these individuals leverage both technological and non-technological means to maintain situational awareness about their condition. We discuss how these practices serve to infrastructure their self-management routines, including medical treatment, diet, and glucose measurement-monitoring routines. Our study provides insights into adolescents’ and young adults’ lived experiences of using HCL systems and related technology to manage diabetes, and contributes to a more nuanced understanding of how the HCI community can support the contextualized management of diabetes through technology design.

## 1 INTRODUCTION

Type 1 diabetes (T1D) is an autoimmune disorder most commonly diagnosed in young people, displaying a notable rise from an estimated 1.48 per 1,000 individuals aged 19 or younger in 2001 to 2.15 per 1,000 in 2017 [45]. T1D is characterized by inadequate endogenous insulin production [48], resulting in persistent hyperglycemia (i.e., high blood glucose/sugar levels) and necessitating lifelong exogenous insulin administration to counter this effect [51]. T1D is also recognized as among the most psychologically and behaviorally intricate of all chronic medical conditions (e.g., [36]). Managing T1D involves attending to and exerting control over complex and dynamic aspects of personal life (e.g., diet and activity) and administering medication day and night [17, 29, 47]. The highly situated nature of managing T1D suggests that there are various individual differences impacting management (e.g., [17]) and no one-size-fits-all therapeutic approach [5], especially for young people [24].

Consequently, self-management practices significantly influence T1D treatment outcomes, as individuals are responsible for 95% of lifelong diabetes management [36], including maintaining vigilance over glucose levels, regular administration of doses of insulin (referred to as “bolusing”) to offset blood glucose increases caused by meals [74], and making adjustments for hypoglycemia (i.e., low blood glucose/sugar levels) or hyperglycemia (i.e., high blood glucose/sugar levels). Because T1D is often diagnosed during youth or adolescence, the age and developmental stage of diagnosed individuals offers an appropriate window for interventions to enhance self-care behaviors [20]. However, adolescents’ and young adults’ (AYAs’) self-management levels notably lag behind other age groups [20], resulting in severe consequences like hypoglycemia and diabetic ketoacidosis (DKA), a critical and acute complication in which the body produces an overabundance of blood acids (ketones) and often results in hospitalization [23]. Hence, swiftly and effectively performing, adapting, and adjusting daily self-care practices [34, 54], referred to as *T1D self-management routines*, are imperative for optimal glycemic control (i.e., maintaining blood glucose levels within the targeted range).

Technological support can be crucial for T1D self-management routines of AYAs, with context-aware diabetes technology like hybrid closed-loop (HCL) insulin pump systems playing a pivotal role. HCL systems provide comprehensive situational information to individuals, including near-future glucose patterns and visualized glucose readings. These integrated systems combine continuous glucose monitors (CGMs, which can continuously monitor glucose levels and update values every five minutes), insulin pumps or CGM receivers (e.g., mobile apps displaying glucose trends), and predictive algorithms (e.g., [5, 51]). Within HCL systems, algorithms facilitate automatic insulin adjustments and suspensions based on glucose predictions [62], issue alerts for glucose fluctuations (e.g., [29, 55]), and assist in maintaining bolusing routines, offering various advantages. Such complex functionalities clearly support young people’s awareness about their conditions, but these technologies also require incorporation into a broader set of resources employed for self-management—and learning how to infrastructure routines that can leverage these resources effectively.

People learning self-management routines with HCL systems have been shown to encounter disruptions or breakdowns, such as alarm fatigue and abandonment of use [51]. Consequently, young patients also seek awareness support from other aspects of their daily lives, such as bodily cues [19] or self-experience [17]. Taken together, this personalized combination of technological and non-technological resources and the AYAs’ relations to them that are embedded in different temporal and social contexts constitute a health *infrastructure* that—when working well—invisibly supports the everyday routines of self-care [61, 65, 66] and facilitates learning of proactive management techniques [52]. In this case, the situational awareness gained by these infrastructures’ use facilitates proactive blood glucose management, leading to improved overall glycemic control [52]. Thus, it is important to understand how young people assemble and employ both technological and non-technological resources when learning self-management routines amid disruptions to achieve optimal glycemic control.

Prior studies in the HCI community have contributed to understanding contextual factors like personal experience [17] and culture [73] impacting diabetes management, novel technology design for reflection [50], and challenges encountered during life transitions of young adults with T1D [41] using insulin pump technology [37]. However, each of these investigations focuses on specific components of the health infrastructure surrounding T1D self-management; we take the view that AYAs learning to manage this condition learn to use different facets of the infrastructure in different ways and under different conditions (e.g., when breakdowns occur with the HCL technology or when personal experience falls short.) There is still more to be understood about this process using an infrastructural inversion approach [9], specifically exploring how the lived experiences and strategies of self-management draw on both technological and non-technological contexts. AYAs provide a particularly compelling case study for this work given the developmental and social challenges they face in learning these routines, as does the deployment of increasingly automated diabetes management technologies—HCL systems—as a cornerstone of this health infrastructure.

To achieve the goal, we ask: **how do adolescents and young adults infrastructure their resources (i.e., situational awareness) to learn T1D self-management routines?** To answer this question, our study employs semi-structured interviews [60] with AYAs using HCL systems for T1D management. We then conduct thematic analysis [10] to make sense of how these individuals infrastructure various resources—particularly, those that contribute to situational awareness—when learning self-management routines. We finally discuss how AYAs use these infrastructures to support three key self-management routines: medical treatment, diet, and glucose monitoring. Our study’s contribution is threefold. First, we uncover diverse situational awareness in T1D self-management and highlight AYAs’ infrastructuring practices for learning self-management routines when facing breakdowns. Second, we offer nuanced insights into contextualized diabetes management that has been advocated by the HCI community, particularly extending this understanding to younger individuals than have been studied previously. Third, our study informs future context-aware diabetes technology design, which is particularly relevant, as this technology is rapidly evolving [5].

## 2 BACKGROUND AND RELATED WORK

### 2.1 Context-aware technology for health and wellness self-management in HCI

In HCI, personal informatics studies highlight the significance of capturing contextual information with self-monitoring technologies for effective health/wellness self-management. Missing this contextual information can lead to misinterpretation and reduced self-reflection opportunities, which may undermine bodily sense-making (e.g., [2, 14, 53]). These contexts include but are not limited to disruptions in life (e.g., pregnancy [53]), (non-)routine circumstances [14], historical data [32], and relevant contributors to a health condition [59].

Contextual information resources provided by these technologies or systems can aid patients to learn “what is going on around you to decide what to do” [26, 32], which is referred to as *situational awareness*. Situational awareness is particularly crucial for dynamic fields like healthcare management with high information flows and potential consequences for poor decisions [32]—including management of T1D.

However, scholarship on context-aware technology in HCI predominantly centers on personal wellness systems (e.g., designing ambruptive systems [46] or wearable devices [43] to aid in keeping healthy habits), with limited attention to clinical treatment contexts (e.g., [32]). Our study investigates HCL systems, an increasingly utilized clinical treatment technology leveraging physiological sensor-based data, and our larger aim is to contribute insights for enhancing the integration of contextual information for effective situational awareness in T1D management.

### 2.2 Contemporary technology for diabetes care

Effective T1D management entails substantial responsibilities, including frequent glucose monitoring, precise carbohydrate quantification, and insulin administration [62]. In addition to these tasks carried out at all ages, young people also navigate changes and challenges influencing treatment routines, such as physical growth and sexual maturation [24], time-related conflicts [19], and peer influence [8], among other considerations. These responsibilities and challenges increase the burden of diabetes management [24, 62].

To alleviate the management burden [62], there has been rapid evolution in diabetes technologies in recent decades (e.g., [5, 51]). Studies have reviewed novel and emerging diabetes technologies (e.g., [5, 62]), primarily encompassing insulin pumps (wearable digital devices delivering basal or bolus insulin subcutaneously, often with tubing), continuous glucose monitors (CGMs, comprising of a receiver for glucose values, a transmitter, and a subcutaneous glucose sensor) [5], as well as open- and closed-loop systems. Open-loop systems include sensor-augmented pumps or SAPs, which integrate CGMs and insulin pumps, requiring manual management. Some SAPs include a feature for automatic insulin suspension to avert hypoglycemia [5]. More advanced hybrid closed-loop (HCL) systems, also called automated insulin delivery (AID) systems, are comprised of CGMs, insulin pumps, and, increasingly, mobile applications. HCL systems incorporate predictive algorithms that can automatically adjust insulin delivery and suspend insulin delivery based on algorithmic prediction of glucose values [62]. Such systems also include alerts or alarms notifying individuals about various situations, such as high or low glucose levels, low battery of diabetes management devices, and low insulin storage [63]. Finally, HCL systems enable users to configure various modes (e.g., to accommodate changing insulin needs with exercise), and allow others (e.g., parents) to co-monitor blood glucose levels of the individual managing diabetes via smartphone. Hybrid closed-loop systems and CGMs can lead to promising glycemic control outcomes (e.g., [4, 29]), notably addressing hypoglycemia, a pivotal facet of diabetes management [25], in vulnerable populations like young people.

Given the remarkable pace of advancement in diabetes technology [5] and its practical adoption by adolescents and young adults, it becomes essential to comprehend the ways through which young individuals integrate or employ these technologies to develop personalized management routines.

### 2.3 Learning of diabetes self-management routines by young people

*Routine* refers to patterns of actions that can be performed, adapted, and modified [56, 61]. Self-management routines are pivotal in navigating life with T1D and are adapted when experiencing disruptions, such as during the COVID-19 pandemic [34]. This especially holds true for young people, who undertake an increasing responsibility for self-management in their daily lives [36, 42] and encounter unique challenges associated with social, psychological, and physiological factors impacting T1D self-management (e.g., [19, 24, 36, 58]). Moreover, individual differences in the daily diabetes management process among young people [17], involving dietary choices, physical activity, and school attendance [24, 41], underscore the significance of personalized management over adherence to generic health guidelines [17].

Learning personalized self-management routines, to include learning from glucose data [42] and body signals, acquiring personal experience, and performing carbohydrate calculation [19], is an imperative process for young people, particularly considering that self-management levels among this population are often suboptimal [20]. However, learning a self-management routine can be overwhelming, cumbersome, time-consuming, and disruptive [17], as young people must consider intricate aspects of daily routines and comprehend the correlation between glucose levels and various life experiences [17]. This can be especially significant for individuals undergoing life transitions and events like moving away from home, socializing, attending parties, and enrolling in college [41, 57]. All of these transitions may challenge already-established self-management routines. Inability to adapt to life routine breakdowns or disruptions can result in compromised glycemic control [24] and other consequential risks such as diabetic ketoacidosis (DKA), “a serious acute complication” of T1D [23].

Various aspects of diabetes technologies, as mentioned in Section 2.1, support AYAs’ self-management with T1D (e.g., [52]). Studies have shown that hybrid closed-loop systems can improve patients’ active or passive awareness of hypoglycemia and hyperglycemia (e.g., both during waking hours and during sleep [29, 39, 47, 55]). Recently, Messer and colleagues underscored the importance of situational awareness in signaling AYAs’ proactive blood glucose management in the context of hybrid closed-loop system usage, associated with improved glycemic control compared to reactive management [52]. However, the specific constitution of this situational awareness remains unclear. Additionally, it is reported that young people often experience breakdowns during technology-assistive learning processes, such as alarm fatigue, sensor errors, and other technical issues, as well as emotional factors like diabetes distress [30], leading some to abandon using diabetes technology altogether [51].

In summary, prior research primarily concentrates on specific contexts—either technological or non-technological—regarding young people’s self-management routines or experiences. Few studies have explored holistic approaches to self-management routines spanning both contexts, a perspective that would be particularly valuable in understanding how individuals integrate diverse learning resources when encountering disruptions.

### 2.4 Infrastructuring for health management

#### 2.4.1 Infrastructure and infrastructuring.

Generally, infrastructure encompasses physical (e.g., highways), technical (e.g., e-health systems), information (e.g., social networking sites), and human resources that facilitate a society’s routine functions [61]. In Star and Bowker’s work “How to infrastructure” [66], infrastructure is used as a verb, and it is noted that the human body can also be an infrastructure (e.g., emotions). As indicated in Star and Ruhleder’s work [67] and summarized by Dillahunt and colleagues, infrastructure is “relational, situational, and practical” [21, p. 5] and usually invisible or transparent unless (or until) its users experience breakdowns or disruptions [35, 61, 67]. In other words, infrastructure is not merely a passive substrate but also embodies an enduring process of alignment between individuals and their contextual settings [61]. In the course of this process, the actions involving aligning and navigating infrastructure are referred to as “infrastructuring” [27, 66]. Individual or collective infrastructuring practices typically aim for specific goals [27]. In the HCI community, infrastructuring has been adopted to investigate civic participation [44], community-centered mentorship [21], mobile knowledge tasks [27], resilience [61], creativity work [64], and healthcare (e.g., [7, 35, 71]), with a focus on emphasizing the interactions and arrangements involving diverse technological elements and human actors [21]. As the focus of the present study is T1D management, we next expand related studies centered around health management and healthcare.

#### 2.4.2 Infrastructuring in health management contexts.

In the HCI community, the infrastructuring lens has often been adopted to understand individuals’ practices of aligning or assembling various entities when experiencing breakdowns or uncertainties during interactions with healthcare systems (e.g., [7, 18, 35]). For example, Bhat and colleagues [7] examined how patients and healthcare professionals aligned and coordinated technologies as diverse as WhatsApp to continue healthcare services and consultation (i.e., infrastructuring work) during the initial COVID-19 pandemic disruptions, specifically in the context of telehealth. Gui and Chen [35] focused on macro-level dynamics of the healthcare system, such as healthcare providers and insurance companies. Their study shed light on how caregivers align and navigate the intricate and fragmented healthcare infrastructure, thus optimizing its functionality at the “micro, individual scale.” Furthermore, infrastructuring practices occur during life transitions like gender [71] and motherhood transitions [11], wherein individuals may experience dramatic changes in the body (e.g., hormone and emotional changes) but also systematic infrastructural support deficiencies. For example, Wilcox and colleagues investigated the care infrastructuring of transgender and non-binary people amid various uncertainties in society (e.g., unpredictable consequences of exposure under misinformation or harmful information related to gender discourses) [71]. They found that this population often infrastructures their care by “assembl[ing] together components of informal, digital social worlds, formalized knowledge sources and processes, and self-reflective experiences” [71, p. 8], such as incorporating self-tracking technologies to make sense of emotional changes.

The diagnosis of T1D disrupts young people’s relations with their body, rendering visible the previously backgrounded processes and routines by which the body cares for its own blood glucose levels [67]. These changes manifest in everyday activities impacting blood glucose levels such as eating, drinking, walking, driving, sleeping, and working, which necessitates the assemblage and integration of new kinds of health infrastructures. Until truly formalized and learned, these infrastructures can be brittle and prone to failure; for example, James and colleagues [41] comprehensively outlined breakdowns in the transition to university life for young adults with T1D. These breakdowns, reflected in uncontrolled glucose levels, range from eating (e.g., exposure to new food) to drinking (e.g., increasing social events among young people and the introduction of alcoholic beverage consumption) to physical activities (e.g., walking to school and house cleaning) to uncontrollable schedules (e.g., ad-hoc meetings). Through adopting the lens of infrastructuring, our study examines how AYAs employ various resources, both technological and non-technological, to learn self-management routines.

## 3 METHOD

Our study employs a semi-structured interview method [60], as we wanted to qualitatively and empirically understand patients’ lived experience with managing T1D in the context of a HCL insulin pump system–and, in particular, to investigate the strategies employed to manage blood glucose fluctuations based on the afordances of the HCL user interface.

Adolescents and young adults (AYAs, aged 15–25) with T1D exhibit the highest average HbA1c levels compared to other age groups [31] and share distinct developmental, cognitive, and psychological transitions [1, 16, 38, 52]. Common challenges with effective self-management may be exacerbated by the unique transitions and hurdles encountered by AYAs. These transitions include but are not limited to physiological changes (e.g., physical growth and sexual maturation [24]), the shift to independent living (e.g., moving to college) [41], social engagements [41], mental well-being [6], and peer influences [8], all of which can disrupt established management routines. With this in mind, we study AYAs as a cohesive group in this research in order to explore this *developmental milestone*, rather than solely adolescents or young adults. We recruited 20 AYAs (see Table 1 for details) aged 15–25 (50% male, 55% non-Hispanic white) from the patient population at the University of Colorado Anschutz Medical Campus in the United States.

**Figure 1:**
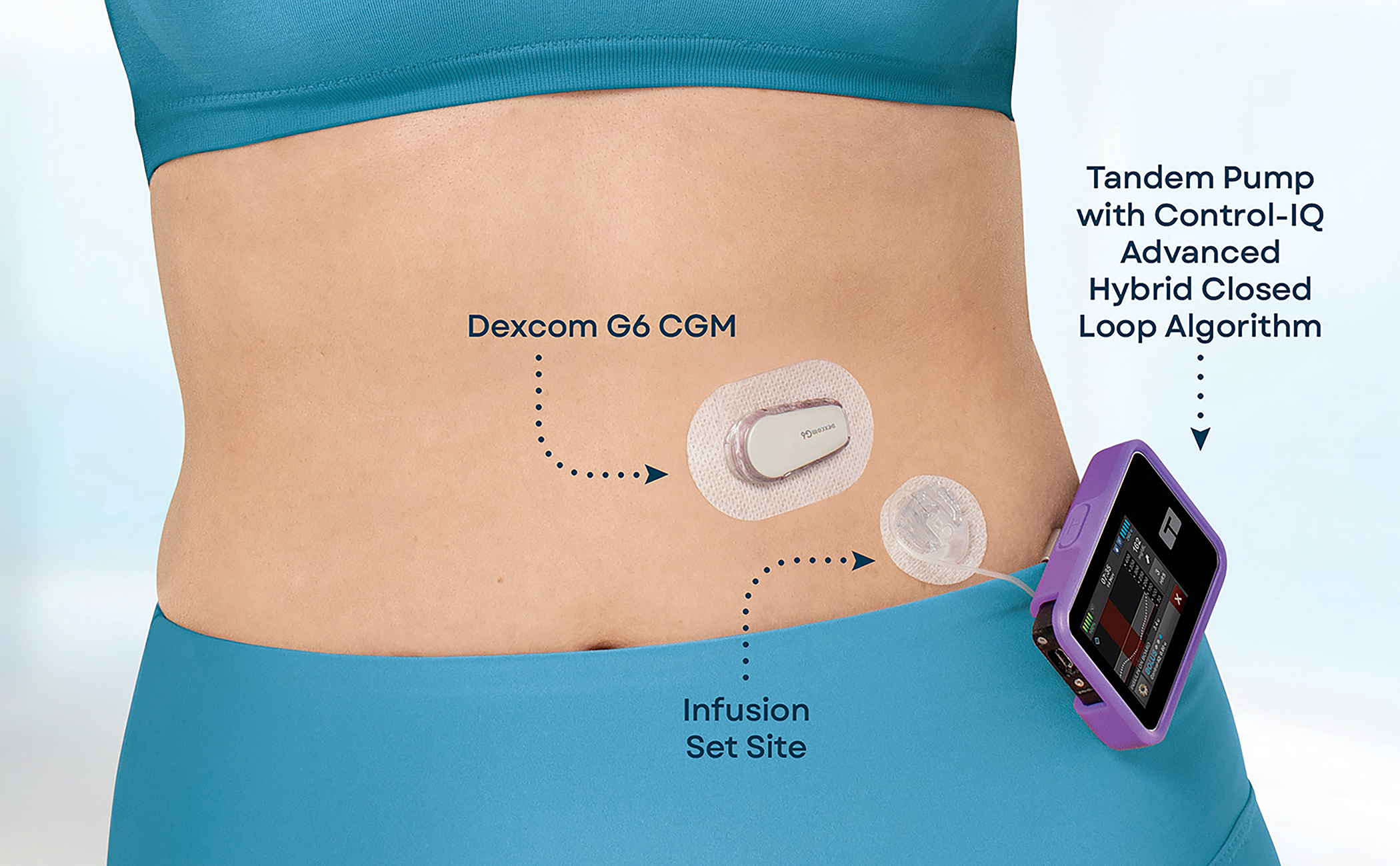
The image shows how an individual usually wears a HCL system (i.e., a CGM along with an insulin pump with an algorithm). The CGM senses glucose levels and sends data to the insulin pump via Bluetooth. The insulin pump shows glucose numbers and predicted trends and delivers insulin to the body. The algorithm embedded in the insulin pump predicts glucose levels and adjusts insulin delivery accordingly.

All participants were using an advanced HCL system for T1D management (see Figure 1), specifically the Tandem t:slim X2 insulin pump with Control-IQ technology (referred to Control-IQ in our study) paired with a Dexcom G6 CGM, prior to study enrollment. The Control-IQ algorithm predicts glucose levels 30 minutes in the future using data from the Dexcom CGM and the device’s insulin delivery history and adjusts insulin delivery accordingly [28]. This includes dynamically adjusting basal insulin and also delivering an automatic correction bolus of insulin up to once per hour to maximize in-target glucose levels (70–180 mg/dl).

After consenting to the study, participants completed a virtual enrollment visit with a study coordinator or investigator. Information was collected per participant report on diabetes history, diabetes technology use, and demographics. Participants were instructed how to complete a user-initiated, real-time survey each time they interacted with their insulin pump for a period of 2 weeks. The surveys were designed to collect data on what prompted the user to interact with their pump (i.e., an alert from the device, habitual checking, or symptoms of hypoglycemia). For the purposes of this study, the survey served as a memory cue for the participant and allowed the interviewer to ask specific questions prompted by each participants’ specific actions over those two weeks. Moreover, we consider participation in the survey component of the research to be a structured activity intended to help the participants reflect more explicitly on their diabetes management practices.

At the end of the 2-week study period, participants completed a final virtual interview with the study coordinator or investigator. Interviews lasted from 15–45 minutes (mean = 35 minutes), depending on the responses of the participants. A series of predetermined questions were asked to participants during the interviews. Questions were open-ended and designed to foster a conversation about diabetes management, both over the 2-week study period and over the participant’s entire experience with diabetes. The topics of these questions centered around reasons for checking and managing blood glucose, self-management routines with HCL systems, atypical or unusual activities disrupting participants’ routines, and reasons for perceived (un)successful self-management.

Our study was approved by the IRB at the University of Colorado Anschutz Medical Campus. Several trained clinical professionals on the research team possess credentials aligned with accepted medical ethics guidelines for working with patients. All interviews were conducted and recorded via Zoom. Data collection was completed after the interviews. Transcripts of the interviews were then generated using the Otter.ai platform^1^, then deidentified by the study coordinator prior to analysis.

We conducted thematic analysis to comprehend how our participants infrastructure resources and maintain situational awareness in order to learn how to manage their condition. Four authors on our research team open-coded eight randomly selected transcripts. We then identified similar patterns among codes and inductively clustered descriptive codes into sub-patterns using a Miro Board^2^. Two high-level themes were synthesized, highlighting participants’ strategies of maintaining diabetes awareness, from the clustered sub-pattern themes. During this inductive clustering process, our full research team met weekly to discuss and iteratively refine sub-patterns and themes, ultimately forming a codebook for deductive coding (see Table 2). Utilizing this codebook, two authors independently performed deductive coding on the full set of 20 transcripts and created coding memos to develop the study’s narrative. With respect to understanding and interpreting the data from this study, we note that our research team represents both lived experience with T1D and clinicians with years of experience working with T1D patients.

Our research team does also include one diabetes research clinician who took a full-time position with Tandem Diabetes Care, Inc. following the data collection phase of this project and one certified pump trainer for Tandem devices. These affiliations with Tandem provided our team with additional expertise on interpreting participant comments about the operation of the specific device described in this study.

## 4 FINDINGS

### 4.1 Reexamining relations with health infrastructures: Maintaining diabetes awareness without technology

#### 4.1.1 Embodied manifestations.

Embodiment typically refers to the connection between mind and body, and how our senses through our bodies inform our experience; as such, the field of embodied computing investigates the connection between our body’s experiences and technology (e.g. through wearables or medical technology devices) [22]. One aspect of our findings involved how participants’ senses influenced their awareness and subsequent interactions with the technology. Most participants (13/20) reported that they recognize blood glucose status from body signals and other embodied feelings caused by blood glucose fluctuations, which suggests the importance of self-awareness for learning management routines such as when to engage with their insulin pump and/or CGM receiver. These manifestations can be physically and/or emotionally embodied and are more easily detected during hypoglycemia, as advised by our clinical team.

Nearly half of these participants who mentioned embodied awareness (5/13) reported recognizing their blood glucose levels through physical symptoms, notably hypoglycemia-related shakiness and nausea, prompting them to monitor their diabetes devices and subsequently make informed treatment choices. As P10 described their awareness, *“I might check [my blood sugar on the insulin pump] if I was [. . . ] feeling particularly nauseous or shaky or something.”* P2 echoed such feelings: *“It feels like shaky legs [are] kind of my main thing, like my legs start to shake a little or feel funny and like kind of weird to walk or move then it starts to or I start to feel off. ”* Some participants used such signals as a means to proactively manage blood glucose levels. As P11 shared, *“I just get a little shaky. And that’s about it. I generally catch it before anything happens.”*

In addition to these common symptoms, participants also report awareness of low blood sugar levels (hypoglycemia) from other symptoms, including sweating, dizziness, and headaches. P2 described somatic feelings such as hunger and tiredness associated with hypoglycemia.

However, embodied symptoms of hyperglycemia might not be as reliable as those of hypoglycemia. When present, these symptoms (e.g., feelings of tiredness or weakness [70]) can also signal diabetes device users to validate their glycemic status by checking their CGM, either on their insulin pump or by using a phone application, particularly with uncertain symptoms related to hyperglycemia.

“I’m like laying down or sitting down and I start to become like. . . suddenly really hot and kind of sweaty and hungry, then I also think it’s low. When it’s high, it’s hard to tell. . . I usually just become really tired, really fast. And then I think, maybe it’s my blood sugar, and then I’ll check, but sometimes it’s not. I’m just tired.” (P2)

Furthermore, participants acknowledge glycemic status via emotional manifestations. As shared by P10, beyond physical signals of nausea and lethargy, *“I also feel like I’m kind of more irritable when I’m high.”* P20 also shared the symptoms of annoyance and frustration in detail while interacting with clients in the workplace, particularly during hypoglycemia:

“I don’t think I have much of an emotional reaction unless it’s like a very severe low. I’ve had situations with the client. I think I first ignored it because I was like. . . okay I’m in session away. I then ignored it again. And then I turned off my insulin—like I paused all insulin. The third time it beeped at me it was like 65, with double arrows going down or something. And so I was like. . . I was feeling sorry. I was starting to get frustrated with the client. Because that’s what happens when I get low blood sugars, then I start getting frustrated with people. It’s more of an annoyance than frustration. It’s actually I’m just annoyed because I’m like, ‘Don’t talk to me.’” (P20)

#### 4.1.2 Food information.

As indicated by participants, objective dietary details, especially carbohydrate amount, hold significant importance in acquiring essential awareness for managing daily routines. This is particularly relevant for routine bolusing, intricately linked with blood glucose fluctuations (e.g., pre-meal insulin dosing).

Participants noted how clear or obvious food information made it much easier to calculate carbohydrates for optimal bolusing, leading to improved blood glucose level control. As P8 stated, *“for breakfast this morning, I got an egg sandwich from Starbucks. And so I know, like, that’s very obvious what the carbs are. It says it [carbohydrate quantity] right on it. And so that’s why I didn’t like go high.”*

Simultaneously, many participants expressed concerns about incorrect calculation that was the suspected cause of high or low blood glucose levels. As P8 shared, *“part of the reason I went high [was that] I just also way underdosed for what I ate, which I know that I did that. And so that also is why I went high is I got a plate of pancakes and I only dosed for 25 carbs.”* P7 echoed with similar experience: *“I tried going low carb, but then. . . But then I apparently carb counted incorrectly. So then I went back up high. That’s been a bit of an issue, like trying to carb count exactly correctly.”*

Such scenarios may arise from ambiguous food information, especially in contexts like dining out that are different from their daily diet routines, where individuals have to learn to estimate carbohydrate quantities based on limited information. Such estimations can be inaccurate when dealing with unfamiliar food(s). As P10 stated, *“we went out to dinner, and there was Mexican food, and I definitely underestimated how many carbs there [were].”* As such, participants commonly develop a pattern of learning daily bolusing routines through estimations based on general food types rather than specific meal information. They are often making estimates (which as noted in examples above, may sometimes be inaccurate), and some participants like P20, who said *“I’m an estimator,”* had a good enough sense for certain foods that they often didn’t check nutrition information.

However, unlike estimators, several participants adopt a precise calculation approach. For instance, P14 reported weighing food in cases of absent labeling: *“We have a little scale that can. . . like. . . weigh stuff out that doesn’t come with a nutrition label: apples or grapes or something.”* In summary, participants adopt a variety of approaches to using the information available to them about food in order to influence their diabetes management decisions.

#### 4.1.3 Self-reflective patterns.

We also note that participants’ awareness of glucose status seems to change over time, stemming from self-acquired knowledge and introspection on lived experience, such as identifying summarized patterns of symptoms or blood glucose fluctuations. Participants described learning when to prioritize glucose monitoring, adjust treatment strategies, and decide on preventive treatments from such self-reflective patterns. It is worth noting that self-reflective patterns involve participants’ reflection of subjective experiences, while food information primarily pertains to objective data (e.g., carbohydrate quantity) as previously discussed. Additionally, this pattern emphasizes the learning of when or what behavioral effects impact blood glucose fluctuations, distinct from embodied manifestations centered on self-recognition of blood glucose status.

Several participants observed that their glucose levels were usually low during work, as shared by P6 who skipped bolus during work despite creating an intentional relatively high blood glucose levels to avoid hypoglycemia: *“Typically what it is [. . . ] I find that if I bolus I usually have a low during work. So then I have to eat again, which I don’t like doing while working because then I have to kind of interrupt work.”*

Physical activity can also affect blood glucose levels, but that impact varies individually and therefore is often self-discovered. Many participants are aware of a pattern of post-exercise blood glucose fluctuations, which informed their learning of treatment routines. For example, P1 highlighted increased attention to post-exercise blood glucose levels due to their tendency to be low: *“Later that day. I’m guessing I was just keeping an eye on it just because I had been active all day. I have a tendency to crash after exercise.”* As such, some participants learned to build and maintain a new routine from reflection on similar tendency, exemplified by P19’s experience:

“Before exercise, I’m always checking my phone to see if my number’s a good number. And then after because that’s usually when I get all the lows right after. Usually check to see if I need a snack or if I need some fast acting sugar. And then keeping with the routine daily, because it tends to help with the number of highs or lows I have.” (P19)

Relatedly, P13 highlighted a strategy of adjusting bolusing routines preventively in response to possible blood glucose drops after exercise: *“So if I’m planning on going to the gym, maybe I typically will not bolus for a snack, just because I know that I drop when I go to the gym. So I’m just trying to be preventative.”* Moreover, such awareness makes participants learn how to configure insulin pumps in particular contexts, such as at school. As P11 pointed out, due to their exercise routines, they set “exercise activity” mode on their insulin pump (which enables higher and narrower blood glucose targets to accommodate the likely natural drop) as a default mode to prevent being worried about out-of-range glucose levels at school: *“When I’m like doing weights, or something similar, any actual exercise, but I kind of just leave it on during most days because at school, I don’t want to worry about going too low. It just leaves it a little higher than normal.”*

Besides exercise, some participants observed individual-specific daily fluctuations (i.e., out-of-range blood glucose levels at a given time of day). Notably, some daily glucose fluctuations naturally happen. For example, elevated blood glucose levels, known as the “dawn phenomenon,” can occur in the morning due to a natural cortisol spike, leading to insulin resistance and elevated blood glucose [15]. Nonetheless, some participants still need to learn this through daily patterns and understand the personal impact of such natural fluctuations by themselves rather than solely from doctors, similar to the exercise example mentioned earlier. For example, P1 shared a lived experience of challenges in blood glucose level control when waking up with high levels: *“If I wake up high I usually have a terrible time getting it down.”* Conversely, P2 often experienced stable blood glucose levels in the morning and at night, leading them to allocate less attention to these periods. As P2 shared,

“And then there’s times especially when I have school where I don’t have breakfast, like right when I wake up So it usually stays pretty constant because I haven’t eaten anything that will make it go high and then low and I haven’t entered anything or anything. So usually, I don’t check it as much in the morning as in the night. Because it just doesn’t usually seem to be going low or high at bedtime.” (P2)

Furthermore, several participants learned through patterns that getting sick and drinking alcohol significantly influenced blood glucose fluctuations and insulin treatment effectiveness so that they could manage differently “on certain days.” As P4 detailed:

“And usually when I start getting sick, my blood sugars run kind of high, and I usually have to take more insulin to bring them down. [. . . ] Maybe drinking alcohol. I know that I found out in high school that. taking shots and drinking, let’s say vodka, will actually make my blood sugar drop pretty quickly. The first time I ever drank, I actually went very low. [. ] So that’s one thing that I learned and just more of like the habits and like, how my diabetes is on certain days.” (P4)

### 4.2 Reexamining relations with health infrastructures: Maintaining diabetes awareness with technology

#### 4.2.1 Data awareness.

Because blood glucose control is the key component of diabetes management, blood glucose data is a key component of management strategies. This kind of data awareness can aid participants’ learning of daily routines, typically involving real-time visual monitoring of blood glucose status to support in-the-moment decision-making. Data awareness refers to the monitoring, perception, and comprehension of visible data such as glucose numbers displayed on management devices, including CGM monitors and mobile apps connected to CGMs (which continuously monitor glucose levels and update values every five minutes).

Participants highlight how they track when their blood glucose is high or low through regularly checking data. For example, P16 said that *“I just check it [numbers] regularly,”* responding to the question of how to determine high or low blood glucose status. Some participants also prefer visualizing data to comprehend their status due to their affinity for numbers. As reported by P1, *“I just like seeing the adjustments made when it’s automated. I like data, numbers, and stuff. So I like seeing what it [blood glucose] does.”* Moreover, participants often make judgments about the acceptability of their current status through numerical ranges (i.e., blood glucose levels are in normal range or not), from which they can learn when to ignore or take action. As P5 said, *“If my glucose is in check, then I don’t really think about it.”* P5 explained it further, saying that *“I mean it’s at the level it’s supposed to be, then I think about other things. I’d say [if the levels are] somewhere in the hundreds. . . they’re telling me that I need to be at like 120 or something”.*

Notably, participants tend to check data more frequently (and sometimes, as they would describe it, excessively) when their blood glucose levels are out of range, which can lead to negative emotions, such as stress. For example, as P17 shared,

“Yesterday I was around 300 for a really long time. But I was just waiting for it to drop down. And I wanted to, like, keep checking on it and stuff, but it was not productive to be checking on it as frequently as I wanted to. So it would just be stressing me out. [I went] to look at it every, like, five minutes. And obviously, nothing’s gonna happen in five minutes.” (P17)

#### 4.2.2 Nudging.

Our participants are usually aware of their status through technological *nudges*. These nudges, comprising notifications or alerts from diabetes technologies like insulin pumps, continuous glucose monitors, and mobile apps, serve to notify individuals about various situations, such as high or low glucose levels, low battery of diabetes management devices, and low insulin storage [63].

Nudging is indispensable for some participants in their daily diabetes management. Specifically, many participants tend to disregard or overlook their blood glucose levels until prompted by nudges. As P7 shared, they “rely on” such nudges during school: *“Yeah, I rely on it a lot, especially because during school I’m not thinking about oh, like unless it beeps at me then I check it. ”* Consequently, some participants expressed a desire for additional nudges to assist in being aware of their status, particularly for prolonged periods of high or low glucose levels. For example,

“I think if I had more alarms to check my pump, it would have helped more. Because usually I’ll have an alarm like—Oh, your blood sugar is going high. But when my blood sugar is staying high, it will not give me alarms. Yesterday, I was high for a good chunk of time, and like, I wouldn’t have, maybe, one [alarm] and then bolus. And then I wasn’t able to check until I woke up and I was still high.” (P4)

However, participants’ management routines may conflict with other daily routines, such as while driving. In such contexts, participants learned that more accessible nudges could aid timely checking or treatment, helping them navigate technologies in particular contexts. For example, P3 highlighted that phone nudges were more accessible and convenient during driving:

“So if I got an alert while I was driving, I always check in on my phone before I check in on my pump, because usually my pump is in my pocket. And that’s harder to reach with a seatbelt on. But on my phone, like if I’m high, I get the Dexcom alert too. So I get two alerts for being high. But if I’m going low, usually it’s like Control-IQ has predicted your blood sugar will be under 70 in like the next 15 minutes, so it’s just a prediction. So if it’s that then I’ll see just like the t:slim alert for it [. . . ] I’ll just eat a granola bar while I’m driving [. . . ] cuz like my phone I usually put on like the center like in a cup holder or something. Like because I play music off of it. So the first thing I do when I get in cars, I get my phone out so I can figure out what I’m gonna listen to [. . . ] But my pump is more often times than not just in my pocket, but it’s a lot harder to access it from driving.” (P3)

Beyond just being helpful, technological nudges are also often perceived as less obtrusive compared to interpersonal nudges (such as a reminder from a parent). One participant, for instance, highlighted a preference for technological nudges over parental calls to alert them of their glucose levels:

“I prefer to have technology nudges than to have people nudges because even when I was in undergrad, my parents would always ask me [things] like how’s your blood sugar been blah, blah. . . and I would get annoyed and I was like, ‘Stop.’ Or they would call me to tell me to take my insulin and I already did that. Even though I hadn’t done it but like, I prefer tech [technology] nudges versus actual human like calling.” (P20)

However, many participants reported experiencing negative effects from technological nudges, including annoyance and fatigue. For example, P17 thought that alerts can be “overkill”: *“I get really mad when I hear my pump buzzing. It’s just doing its job. I know. But that is so annoying. I guess it can be overkill sometimes.”* P8 detailed such feeling and echoed:

“I’ve maybe got like one alarm when I went high. And so on days like that, the alarms don’t bug me at all, because I’m just doing a good job of like staying in range. But then the days when I’m really out of range all day, I think they’re the most frustrating thing because they’re going off all the time. And so it’s kind of like, on a good day, the alarm sounds like me at all, because I’m not even hearing them. But on the days that I’m higher or lower, then it’s really annoying.” (P8)

Relatedly, P10 expressed *fatigue* and *desensitization* to nudges over time, resulting in reduced attentiveness to alerts. They also noted that *mobile nudges* might get mixed with other notifications, such as text message alerts, potentially reducing their attention capture:

“That [alert and alarms] is really helpful. But I would say I’m definitely a little bit like desensitized to them. So sometimes they’ll just like, you know, keep buzzing and I won’t really notice that much because it’s kind of just a normal thing. Especially like on my phone, because sometimes it’s just, you know, seems like you’re getting a text message or something, and you just don’t worry about it. And then, 20 minutes later, it starts actually beeping at you, and then it gets your attention. I think they’re definitely helpful. But not noticing them is definitely something that’s kind of developed over time.” (P10)

Furthermore, some participants stressed experiencing prolonged instances of nudging even after taking actions in response to them, making them feel stressed. As P17 expressed, *“One of the things that makes me the most stressed. It’s like. . . yeah, I’m already high. There’s nothing I can do about it. And it’s still reminding me. I’m aware. I’m just like, waiting for it to come down.”* P8 echoed: *“. . . If I’m high, it alarms and I give myself a bolus, then it still alarms that I’m high until I actually lower when I’ve already done the action to make a difference.”*

#### 4.2.3 Algorithmic prediction or automation.

Because all of the participants in our study were on insulin pumps with the Control-IQ system, participants generally are aware of near-future trends of glucose levels predicted by algorithms, in addition to aforementioned real-time glucose values. Such awareness is used to learn when and how to proactively engage in treatment. For example, P2 concurrently assessed both factors to determine the need for a snack bolus:

“I bolus when I want to eat, except if it’s low, like 80 or something, and I have a little snack, I don’t bolus them. But I still check [the algorithmic prediction] to make sure. I like looking at the little diamond on the Control-IQ that says if it predicts it will go low, or if it’s orange [indicating that the insulin pump is reducing basal insulin automatically in response to a dropping glucose trend], if it will go low, but not below 70. I like looking at that to see if I can have a snack without entering anything [. . . ] I would probably wait a little bit to see if it changes to red [indicating that the insulin pump is stopping basal insulin altogether because the patient is predicted to go low]. Or if it changes to not go away and it starts going up. And if it starts going up, then I’ll enter [the carbs for] the snack. And if it goes to red and starts going down more, then I’ll eat it, and hope it goes back up.” (P2)

In addition to learning to initiate proactive treatment based on near-future trends, participants also learned when to utilize algorithmic agency for treatment. For instance, P7 noted that algorithmic reliance partially alleviated the burden attributed to manual management in their daily life: *“Especially like especially during sports and everything like that. So I do rely on it a lot and it would definitely be hard like to just manually bolus and like, and it definitely controls [my blood sugar]. And it’s more like, I can rely on it more than my past.”* Such reliance can be attributed to the trust placed in algorithmic agency for routine management. As stated by P12, they believed that their management skills could not surpass the Control-IQ algorithm, leading them to entrust their care to it:

“I don’t even know if I bolused yesterday, I probably just let the Control-IQ take care of it. It’s what I normally do, so.… I don’t think my skills ever got to the point where I’m much better than Control-IQ. Like I think my diabetes taking care of diabetes skill is about on par with Control-IQ so it doesn’t really change too much.” (P12)

In contrast, some participants found certain aspects of algorithmic correction (i.e., automated treatment for hyperglycemia or hypoglycemia) less effective in specific cases, leading them to adopt a more critical approach towards its use. For instance, P8 mentioned taking manual control over insulin dosing during hyperglycemia due to perceived slowness in the algorithm’s correction process:

“And so I know that Control-IQ, at least my understanding, is [that] it slowly gradually corrects me down. Whereas I’m kind of like, I’d rather just get it all on one swoop sometimes and just go lower. And so that’s why I did the five grams [manual correction bolus] is because I know it will do it eventually. But sometimes I’m like, okay, now I just want to give it all now. And I’m seeing that I’m high. So to me, it seems kind of silly to ignore the fact that I’m high and just let my pump do it gradually, when I could just do it myself and do it faster.” (P8)

## 5 DISCUSSION

**Figure 2:**
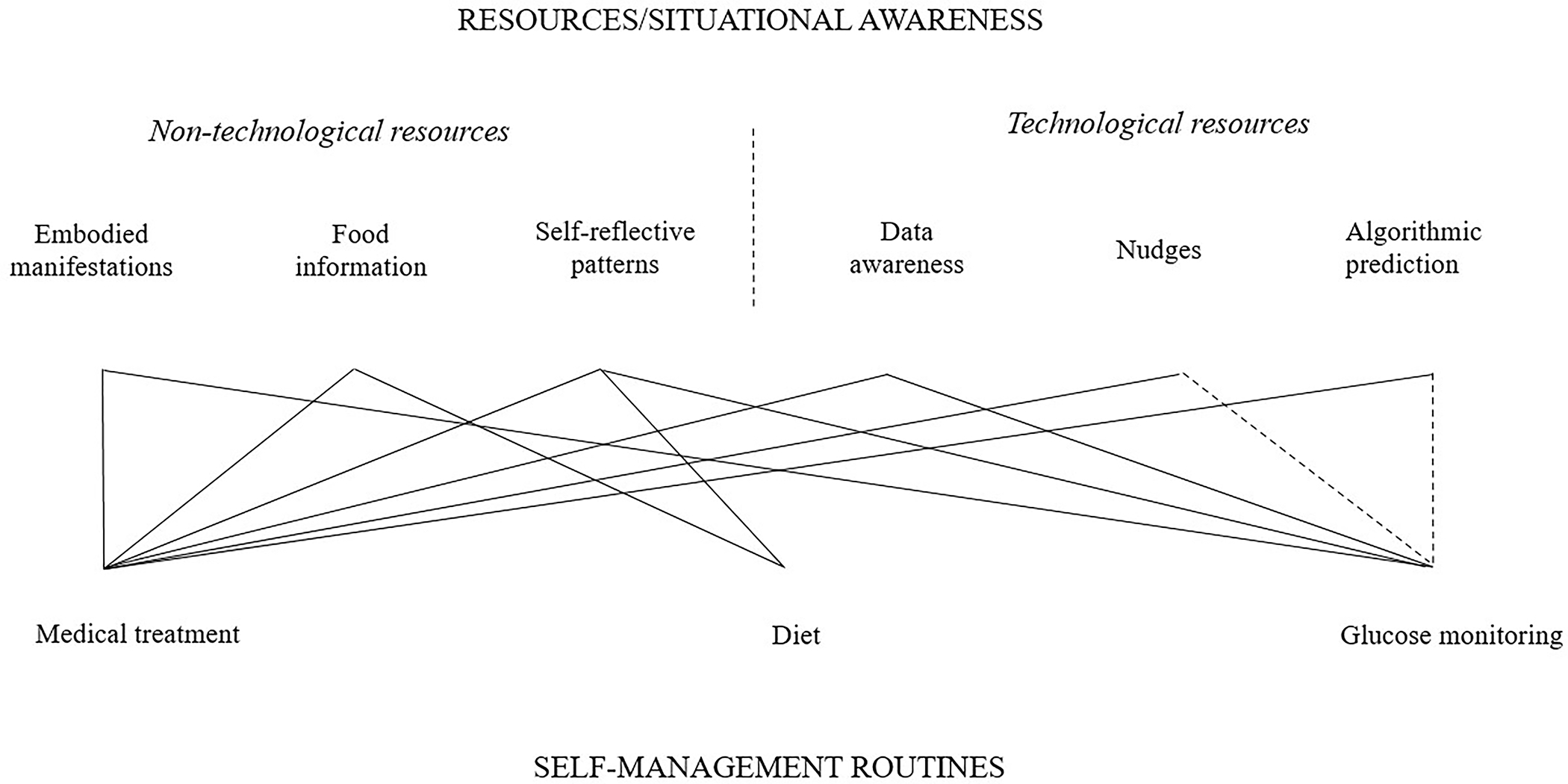
A diagram of how participants infrastructure various resources in support of key T1D self-management routines

Our findings highlight how AYAs with T1D use a combination of technological and non-technological resources to learn self-management routines, primarily through maintaining situational awareness about their condition, including self-awareness signaled by subjective information (i.e., embodied manifestations and self-reflective patterns) and externalized awareness signaled by objective information (i.e., food information, data awareness, nudging, and algorithmic prediction). Our findings also illustrate the importance of situational awareness for AYAs with T1D to aid in the establishment, adaptation, and maintenance of different types of self-care practices. In this section, we discuss how AYAs infrastructure these various technological and non-technological resources to learn diabetes self-management routines embedded in specific practices, including medical treatment, diet, and glucose measurement-monitoring routines, for the purpose of optimal glycemic control (Figure 2).

Like other routines, from grocery shopping to teeth brushing in the morning, self-management requires mundane, yet critical, routines for people with T1D, and because T1D is a chronic illness, these routines must persist throughout their lives [36]. However, unlike other daily routines, T1D self-management routines are significantly more likely to involve infrastructural breakdowns. Therefore, an important but under-studied topic is understanding the *self-organized* [11] *ways of knowing* involved in approaching, identifying, or establishing an individualized diabetes management routine, as individuals’ differences in diabetes management are impacted by “physiological, personal, and social activities” [17]. Simultaneously, AYAs’ rapid biological, cognitive, social, and emotional changes, as summarized by Guo and colleagues [36], suggest various uncertainties for young people managing T1D. In this case, learning from various resources is one of the most prominent processes serving to “support and promote an overarching mechanism” of self-management [19, p. 14].

Prior studies have found that individuals infrastructure different resources to achieve goals of self-care in various contexts, including gender transition [71], telehealth [7], mental illnesses [13, 72], and navigation of the healthcare system, more generally [35]. Our study extends this understanding to T1D, a chronic illness requiring everyday management routines that encompass both medication adherence and careful control over various lifestyle factors. Furthermore, our study enhances understanding in reexamining individuals’ relations to health infrastructure for self-care in the context of algorithm-enhanced HCL systems, providing insights into self-management strategies supported by clinical physiological sensing systems. Importantly, our findings highlight the role of subjective information in building relations with the technological elements of these infrastructures, exemplified by reliance upon bodily signals and insulin pump checking practices, which differ from prior studies on personal experiences without technologies [17], challenges of diabetes technology use [37, 41], and applications supporting self-reflection in the absence of clinical diabetes technologies like HCL systems [50]. Additionally, our findings that re-examine individuals’ relations to health infrastructure suggest an agenda for future research in the T1D context—for example, how to build a more effective self-care ecosystem for young people’s self-management.

### 5.1 Infrastructuring the learning of medical treatment routines

Our findings indicate that our participants infrastructure a range of technological and non-technological resources to learn medical treatment routines (i.e., routines around insulin delivery). Specifically, our study highlights that AYAs learn the timing of reactive and proactive treatment, personalized insulin dosage strategies, insulin pump configuration, the degree that they can (and cannot) rely upon the HCL’s predictive algorithms, and other aspects of informed treatment decision-making by constructing an assemblage of embodied manifestations, self-reflective patterns, data awareness, nudges, and algorithmic prediction.

As previously observed in prior work, adult T1D patients adjust physician-prescribed medication in response to their daily experiences, considering how routine and non-routine activities impact blood glucose variability [17]. Our study expands this finding to encompass algorithmic decisions and nudging, offering additional empirical evidence to describe personalized medical practices within the context of HCL systems. This is exemplified by the routine of manual insulin adjustment as an augmentation to the automated treatment regimen provided by the HCL device. Beyond medication adjustment, we also found that our participants built relations between self-reflective experiences and insulin pump configuration. For example, a participant configured their insulin pump to “exercise activity” mode due to their exercise routine although such setting targets a higher glucose range. Such behavior may be motivated by the fear of hypoglycemia observed among some young adults [57].

As an important component in these infrastructure assemblages, nudging significantly influences the development and maintenance of medical treatment routines, as indicated in our findings. However, breakdowns occur in the learning process due to factors like alarm fatigue, as observed in prior clinical studies on insulin pump and CGM adoption (e.g., [51, 63]). The scenario of alarm fatigue may discourage people from effectively responding to alerts, and in some instances, from using a device at all [51, 63]. Notably, although people can customize alarm configurations on insulin pumps and mobile apps (with the exception of fixed alerts for hyperglycemia and hypoglycemia), certain alerts still evoke feelings of annoyance, excessiveness, and other negative emotions. Our study reveals that alarms may not work effectively in situations where the attention of AYAs to immediate medical treatment is insufficient, such as during sleep, classes, and driving, aligning with Harper et al.’s findings [37] on the inconvenience of alarms during driving among older adults with diabetes. In such contexts, reactive medical treatment (i.e., increases or pauses in insulin dosing) cannot be avoided, requiring more glance-able platforms for signaling their status, such as mobile apps or smartwatch “complications,” as shown in our findings.

Such findings provide insights into context-aware and fatigue mitigation nudging design in the context of chronic illnesses like T1D that do not offer individuals the luxury to ignore, forget, or skip treatments (e.g., adherence to medication regimens) either over the long-term or when experiencing short-term, non-routine circumstances [14]. Prior studies have suggested the importance of incorporating contextual cues into nudging design by leveraging visual cues [43], timed reminders [2], and naturalistic ambruptive systems [46], among others. However, most of these studies focused on context-aware systems for self-*wellness* management. Because of the severe consequences resulting from lapses in effective self-care with T1D, there is a much higher bar for nudging design in the case of diabetes technology. Furthermore, the specific details of the infrastructure assemblages that people with diabetes employ impacts these design considerations: some young people may experience impaired hypoglycemia awareness (i.e., have fewer symptoms of hypoglycemia) [49], which may necessitate different nudging for medical treatment routines. Consequently, we recommend future research to examine variations in nudging designs, and to examine how to enhance the effectiveness, accessibility, and flexibility of nudging across different contexts (e.g., driving, exercising, sleeping, or attending classes) through incorporating individuals’ experiential information. For example, future design might consider the application of various nudging styles from the existing literature, such as naturalistic and/or ambruptive systems that provide visual cues while mitigating alarm fatigue [46].

Importantly, our findings also highlight an important pattern of *proactive* medical treatment (i.e., insulin dosing or pausing insulin dosing without alarms), as exemplified by our findings of responding to algorithmic prediction, embodied symptoms, and checking numbers frequently during certain days or times of day due to self-reflective patterns of anticipated glucose fluctuations. Our findings highlight the HCL algorithm’s role in managing medication routines, exemplified by young individuals’ willingness to cede treatment control to the algorithm in specific contexts. Nevertheless, we also identify challenges in algorithmic predictions, including issues of trust in algorithms and unmet efficiency expectations, aligning with Messer and colleagues’ findings [51]. When these algorithm-related breakdowns occur, participants revert to manual treatment methods. Such breakdowns associated with efficiency indirectly reflects Burgess and colleagues’ conclusions suggesting the efficiency of AI-supported clinical treatment is “a complex, patient-specific question” for adult patients managing the (distinct but related) condition of Type 2 Diabetes [12].

Indeed, contextualized and personalized, algorithm-assisted treatment appears to enhance diabetes self-management for many users of diabetes technology. Messer and colleagues [52] found that proactive pump engagement facilitated by situational awareness (more intuitive rather than factual knowledge) correlates with better glycemic control. Our study expands on these prior findings by offering empirical evidence of how young people incorporate bodily cues, sensations, and self-reflective patterns into their health infrastructure, shedding light on the potential mechanisms underlying improved glucose control through *situational awareness-supported proactive treatment*. In addition to non-technical situational awareness, our findings also highlight the significance of the technological components of these infrastructures in fostering proactive management—particularly algorithmic prediction—by enhancing participants’ awareness of changes in their conditions. Instances in which glucose fluctuations were prevented by, for example, responses to a combination of embodied cues and algorithmic prediction indicate that AYAs assemble or combine multiple awareness-related resources in support of their medical treatment routines. Prior studies suggest incorporating people’s qualitative experience, rather than solely quantitative blood glucose measurements, into medication treatment (e.g., [17]). Our study provides support and actionable context for this suggestion with nuanced insights about what cues young people usually use in everyday self-management—insights that may support future algorithm-assisted diabetes technology research and/or design.

Nonetheless, integrating multiple-source data into algorithm learning systems is a challenging endeavor [33] since bodily cues may be difficult to quantify and can vary between hypoglycemia and hyperglycemia. Subsequent research could consider incorporating additional kinds of contextual information (e.g., self-reflective patterns and biological indicators) for personalized glucose predictions to enhance algorithmic systems—for example, exploring the viability of utilizing biological sensing such as tremor detectors [69] to identify experiential bodily sensations such as reported feelings of shakiness.

As previously noted, trust significantly influences AYAs in acquiring and maintaining algorithm-assisted medical treatment routines. Our research reveals diverse treatment approaches linked to varying levels of trust in algorithms, even encompassing controversies. For instance, some participants rely on algorithm-controlled treatment regimens due to the perceived superiority of these devices’ management capabilities. These findings align with Tanenbaum and colleagues’ investigation of algorithm trust among adolescents and adults towards diabetes technology [68]. However, their study also addresses potential risks tied to excessive reliance on algorithmic treatment in the constitution of health infrastructures, including diminishing self-management skills and reduced vigilance over time [68]. Future research could further explore the impact of young individuals’ trust in algorithms on routine acquisition and the balanced integration of human and algorithmic *agency* to formulate contextually optimized management strategies.

### 5.2 Infrastructuring the learning of dietary routines

Following the diagnosis of T1D, individuals’ relations to food information infrastructure suffer regular breakdowns, largely because food label creators’ relationship with food labels differs from that of people with T1D. Factors such as diet composition, portion size, and timing of food all drastically affect how food intake alters blood glucose. Our study underscores how young people commonly employ self-reflective patterns and utilize food information to establish dietary routines, encompassing timing, food choices, and carbohydrate estimation for specific contexts, such as pre-meal bolusing, exercise, and driving. Prior studies observed that adults with T1D frequently cited dining out and travel as unusual activities requiring adaptation due to difficulties in food intake control (e.g., [17]). Our findings also highlight such difficulties and extend the understanding through identifying the specific infrastructural components that individuals employ to learn a new dietary routine when experiencing disruptions (e.g., using a scale to weigh foods and categorizing food types for estimating carbohydrate amounts). Such active learning processes may stem from routine breakdowns impacted by the dynamic living situations of AYAs, such as challenges associated with living away from home and college social life [41, 57].

AYAs with T1D encounter unique challenges related to their living and social situations, including novel food consumption, social events, and drinking [41, 57]. These challenges have an impact on their glucose control and evoke negative emotions, such as fear, stress, and frustration associated with hypoglycemia [57]. Despite the crucial role of dietary routines in glycemic control, the existing food information infrastructure may not effectively facilitate objective knowledge for people with T1D, as exemplified by our study. While many individuals rely on self-reflection and learning from past experiences through a “trial and error” approach [19], we emphasize the importance of external food information infrastructure and the future design of context-aware diabetes technology for daily management. Although self-understanding is valuable [17, 50], we argue that the design and development of external food information infrastructure can enhance the effectiveness of self-management. This is analogous to navigating through a forest: while it’s possible to estimate a route, following signs (infrastructure) can reduce risk and increase efficiency. However, it is essential to acknowledge the challenges in establishing a systemic food information infrastructure, including factors like ingredient complexity and portion estimation at restaurants.

AYAs, recognizing the dietary self-management challenges that they face, may seek increased support from technological components of their health infrastructure, specifically HCL systems, to enhance their self-management routines. Similar to the findings of Harper and colleagues’ study, which indicated the desire for age- and context-specific enhancements to insulin pumps based on responses from adults aged 45 to 54 (e.g., transmitting voice alarms to earbuds) [37], our study extends this understanding of age-specific customizations to include diet management support for AYAs. For instance, some participants in our study requested nudges or insulin pump configurations tailored to situations like alcohol consumption, which can lead to hypoglycemia. We recommend that future research consider these lived experiences in the design of insulin pumps, focusing on the refinement of context-specific dietary nudges.

### 5.3 Infrastructuring the learning of glucose measurement-monitoring routines

People with T1D experience breakdowns in relations with the body as infrastructure everyday. Our study highlights the importance of assembling resources to learn glucose-monitoring routines (i.e., monitoring numeric blood glucose measurements) in achieving glycemic targets for many people with T1D [25]. Our participants learn when to allocate more or less attention to their blood glucose and when to react with interactions with their insulin pumps from an assemblage of self-reflective patterns, data awareness, and bodily cues. For example, young people usually leverage self-reflective patterns to learn when to monitor their blood glucose frequently (e.g., morning or night, pre- or post-exercise). They use data awareness to monitor blood glucose in-the-moment, especially when levels are elevated, and they use bodily cues to proactively monitor blood glucose numbers. Notably, nudges and algorithmic prediction may also aid the learning of glucose monitoring routines; however, our findings indicate participants primarily utilize these resources for reactive and proactive treatment. We therefore noted their potential connections to glucose monitoring with dashed lines in Figure 2.

Our findings provide important insights into AYAs’ self-care work during the transition to independent health management, complementing Kaziunas and colleagues’ study [42] by extending the understanding of care recipients’ self-care strategies using data-centric diabetes technology. Through infrastructuring multiple resources to adjust or maintain glucose monitoring routines, we observed young people’s strategies to find the relationships among their body, data, technology, and experiences, aiming to gain control over their health and wellness. Kaziunas et al’s study [42] showed that when parents remotely monitored their children’s glucose values, they experienced improved multiplicity (e.g., freedom, empathy, and togetherness) through use of diabetes technology. Our findings about how AYAs construct assemblages of resources in support of glucose monitoring routines enrich such understanding by further offering young people’s perspectives. Future diabetes technologies can utilize an assemblage of contextual and experiential data, as shown in our study, to offer stronger, holistic interventions for diabetes self-management that go beyond glucose values alone [42].

Prior studies have also provided valuable suggestions for incorporating contextual information into self-tracking health technologies. For example, Ng and colleagues [53] suggested that self-tracking systems should contextualize data (e.g., sleep and stress data) generated during non-routine circumstances—pregnancy is the case in their study—to improve bodily sense-making and mitigate misinterpretation. However, these examinations are mostly around personal wellness systems rather than clinical treatment technologies based on physiological data [3]. Additionally, distinct from hierarchical goal-setting and reduced routine tracking for mitigating lapses explored previously [59], self-monitoring for T1D is challenging to simplify. Goals of awareness, taking action, and monitoring [59] can be interconnected. Also, reducing routine tracking [59] may overlook vital contributors, as routines like diet, exercise, and sleep significantly influence medication and glucose monitoring. Therefore, the novelty of our study is in extending these prior understandings to algorithm-supported and data-centric clinical treatment systems (i.e., HCL systems) for life-threatening chronic conditions that are rife with ongoing and repeated disruptions. Furthermore, our study highlights how diabetes data tracking is relational and situational for management routines, and how it contributes to the understanding of critical, chronic condition management. For example, lapses in glucose monitoring routines due to reasons like low energy require alternative support for sustained awareness.

These findings can also provide insights for data visualization of clinically oriented personal health technologies, which largely utilize sensor-based physiological data [3]. We suggest that relevant systems should support the visualization of relations from a mix of self-awareness, physiological sensor-based data, and AI-based predictive data, mirroring the complexities of individuals’ health infrastructure assemblages and providing more insightful feedback and critical reflection to patients, particularly young patients during the crucial learning state of bodily sense-making.

### 5.4 Implications for human–machine collaboration in health management

Our study underscores the importance of incorporating diverse data modalities and rebuilding and maintaining patients’ routines for effective human-machine collaboration in managing complex chronic health conditions. Different health conditions require specific resources and situational awareness to adapt and maintain routines in everyday life. In cases like T1D, both non-medical and medical routines significantly influence one other, emphasizing the equal importance of supporting both. For example, Hurley and colleagues [40] proposed continuously sensing physical activity and sleep routines for cardiovascular disorder management, integrating multiple sensing modalities (e.g., wrist and chest sensors) and users’ contextual input (e.g., labeling data) as inputs into personalized machine learning systems. However, current physiological sensing technologies mainly focus on sensing and prediction, with less consideration of dynamic or fluid collaborations between patients and machines. For example, if systems detect non-routine activities, how can systems provide individuals sufficient and effective awareness to adjust other routines for more optimal management? We recommend that future (semi-)automated clinical treatment and health support technologies should extend beyond the body–machine relationship to encompass connections among body, data, technology, experiences, and circumstances (after the network that we presented in Figure 2), eventually facilitating the support of people’s seamless routines in everyday life.

In other chronic conditions like mental illnesses, existing scholarship in HCI and CSCW suggests that people use technology ecosystems for self-management [13, 72] for purposes like seeking help and balancing life and episodes [72]. These findings prompt further exploration of managing comorbidity of T1D, such as mental health challenges of AYAs with T1D. Given that few studies have focused on exploring the self-care ecosystems for diabetes management, future studies can expand on our work to examine the technology ecosystems people with T1D use and their purposes, which might provide valuable insights for researchers, designers, practitioners, and clinicians to better support patients.

## 6 CONCLUSION

In this study, we conducted 20 interviews with adolescents and young adults (AYAs) with Type 1 Diabetes using hybrid closed-loop insulin pump systems for self-management routines. We find that AYAs maintain diabetes awareness both without technology (i.e., embodied manifestations, food information, and self-reflective patterns) and with diabetes technology (i.e., data awareness, nudging, and algorithmic prediction or automation). We further discuss how AYAs infrastructure the learning of self-management routines served by this multifaceted situational awareness. Our study provides nuanced insights into contextualized diabetes management advocated by the HCI community and informs future context-aware diabetes technology design.

## Supplementary Material

Video Presentation

## Figures and Tables

**Table 1:** Participants’ Demographic Information

Participants’ Identifier	Gender	Age	Race	Ethnicity
P1	Male	22	White/Caucasian	Not Hispanic/Latino
P2	Male	16	White/Caucasian	Not Hispanic/Latino
P3	Female	20	Native HI/ Pacific Islander	Not Hispanic/Latino
P4	Female	22	Native HI/ Pacific Islander	Not Hispanic/Latino
P5	Male	15	Asian	Not Hispanic/Latino
P6	Male	18	White/Caucasian	Not Hispanic/Latino
P7	Female	15	White/Caucasian	Hispanic/Latino
P8	Female	21	White/Caucasian	Not Hispanic/Latino
P9	Male	18	Native HI/ Pacific Islander	Hispanic/Latino
P10	Male	18	White/Caucasian	Not Hispanic/Latino
P11	Male	17	Other	Hispanic/Latino
P12	Male	22	White/Caucasian	Not Hispanic/Latino
P13	Female	16	White/Caucasian	Not Hispanic/Latino
P14	Male	15	White/Caucasian	Hispanic/Latino
P15	Male	16	White/Caucasian	Not Hispanic/Latino
P16	Female	16	White/Caucasian	Not Hispanic/Latino
P17	Female	17	White/Caucasian	Not Hispanic/Latino
P18	Female	16	White/Caucasian	Not Hispanic/Latino
P19	Female	15	Black/African American	Not Hispanic/Latino
P20	Female	25	Other	Hispanic/Latino

**Table 2:** Iteratively Developed Codebook

Theme	Description	Sub-pattern	Description	Example
*Maintaining diabetes awareness without technology*	Adolescents and young adults with T1D seek or preserve situational awareness about their conditions with non-technological factors in everyday life.	*Embodied manifestations*	Participants’ bodily senses can influence their awareness and subsequent interactions with the technology.	*“It feels like shaky legs [are] kind of my main thing, like my legs start to shake a little or feel funny and like kind of weird to walk or move then it starts to or I start to feel off. ” (P2)*
		*Food information*	Participants acquire essential awareness from objective dietary details, especially carbohydrate amount, for managing daily routines.	*“We have a little scale that can… like… weigh stuff out that doesn’t come with a nutrition label: apples or grapes or something.” (P14)*
		*Self-reflective patterns*	Participants’ awareness of glucose status stems from self-acquired knowledge and introspection on lived experience.	*“Later that day. I’m guessing I was just keeping an eye on it just because I had been active all day. I have a tendency to crash after exercise.” (P1)*
*Maintaining diabetes awareness with technology*	Adolescents and young adults with T1D seek or preserve situational awareness about their conditions with various features of diabetes technology.	*Data awareness*	Participants monitor, perceive, and comprehend visible data such as glucose numbers displayed on management devices.	*“I just like seeing the adjustments made when it’s automated. I like data, numbers, and stuff. So I like seeing what it [blood glucose] does.” (P1)*
		*Nudging*	Nudges comprise notifications or alerts from diabetes technologies like insulin pumps, continuous glucose monitors, and mobile apps, serving to notify individuals about various situations about conditions.	*“Yeah, I rely on it a lot, especially because during school I’m not thinking about oh, like unless it beeps at me then I check it… ”(P7)*
		*Algorithmic prediction/automation*	Participants acquire awareness of near-future trends of glucose levels predicted by algorithms.	*“I like looking at the little diamond on the Control-IQ that says if it predicts it will go low.” (P2)*

## CCS CONCEPTS

- Human-centered computing → Collaborative and social computing; Empirical studies in HCI; HCI design and evaluation methods.

## Footnotes

1
https://otter.ai/

2
https://miro.com/
